# A Rare Case of Giant Retroperitoneal Dedifferentiated Liposarcoma: A Case Report

**DOI:** 10.7759/cureus.92142

**Published:** 2025-09-12

**Authors:** Yufei Wang, Hui Luo, Wei Ding

**Affiliations:** 1 Department of Anorectal, The Affiliated Traditional Chinese Medicine Hospital of Southwest Medical University, Luzhou, CHN; 2 Department of Operations Management, Luzhou Traditional Chinese Medicine Hospital, Luzhou, CHN

**Keywords:** dedifferentiated liposarcoma, giant, radical resection, retroperitoneal tumor, soft tissue sarcoma

## Abstract

Retroperitoneal liposarcoma is a type of tumor originating from various non-specific organs in the retroperitoneum and is the most common retroperitoneal tumor. Due to its deep location and insidious onset, early clinical manifestations are not obvious, and it is generally discovered only when the tumor is large, causing complications by invading or pressing on nearby tissues and organs.

This article reports a case of a patient with a giant retroperitoneal liposarcoma who underwent radical resection. Postoperative pathological examination confirmed it to be a dedifferentiated liposarcoma. This case details the surgical removal of a giant retroperitoneal dedifferentiated liposarcoma and the successful postoperative recovery of the patient, vividly illustrating the surgical skills and perioperative management strategies required to handle such challenging cases. This case provides valuable surgical experience for managing giant retroperitoneal liposarcoma and highlights the practical significance of a surgery-focused comprehensive treatment in improving the quality of life for patients.

## Introduction

Liposarcoma is a type of malignant tumor composed of adipocytes with varying degrees of differentiation and atypia, and it is among the most common soft tissue cancers in adults [1,2]. Among them, well-differentiated liposarcoma (WDLPS) and dedifferentiated liposarcoma (DDLPS) are the two main histological subtypes [3]. Due to its deep location and insidious onset, many patients have no obvious clinical manifestations, and most present with very large tumors that may invade important blood vessels and organs [4]. Surgical resection is the best treatment option, as this disease doesn't respond well to chemotherapy or radiation therapy and is characterized by a high rate of recurrence and poor prognosis [5].

This article reports a case of giant retroperitoneal dedifferentiated liposarcoma treated with complete resection. This case is characterized by a large abdominal mass, high surgical difficulty, a close relationship with surrounding tissues, and an unclear preoperative diagnosis.

## Case presentation

**Figure 1:**
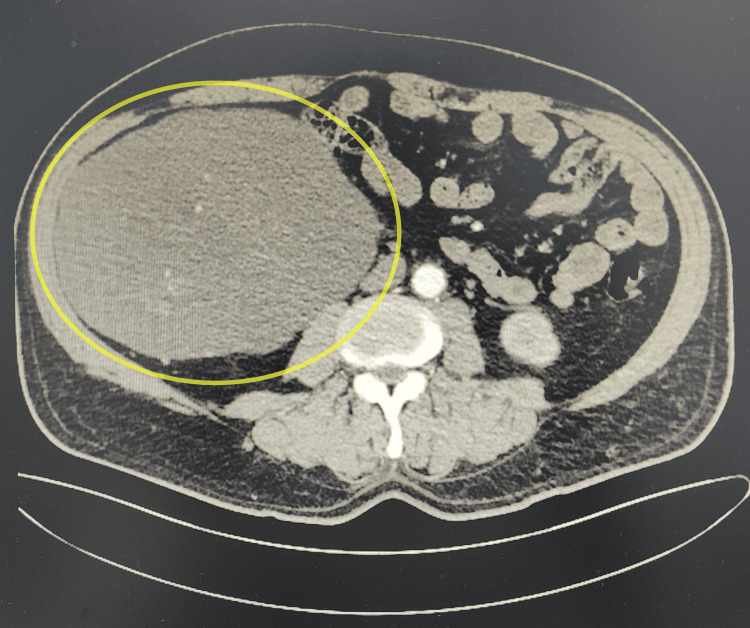
Axial CT scan of the abdomen The yellow circle indicates the retroperitoneal mass.

**Figure 2:**
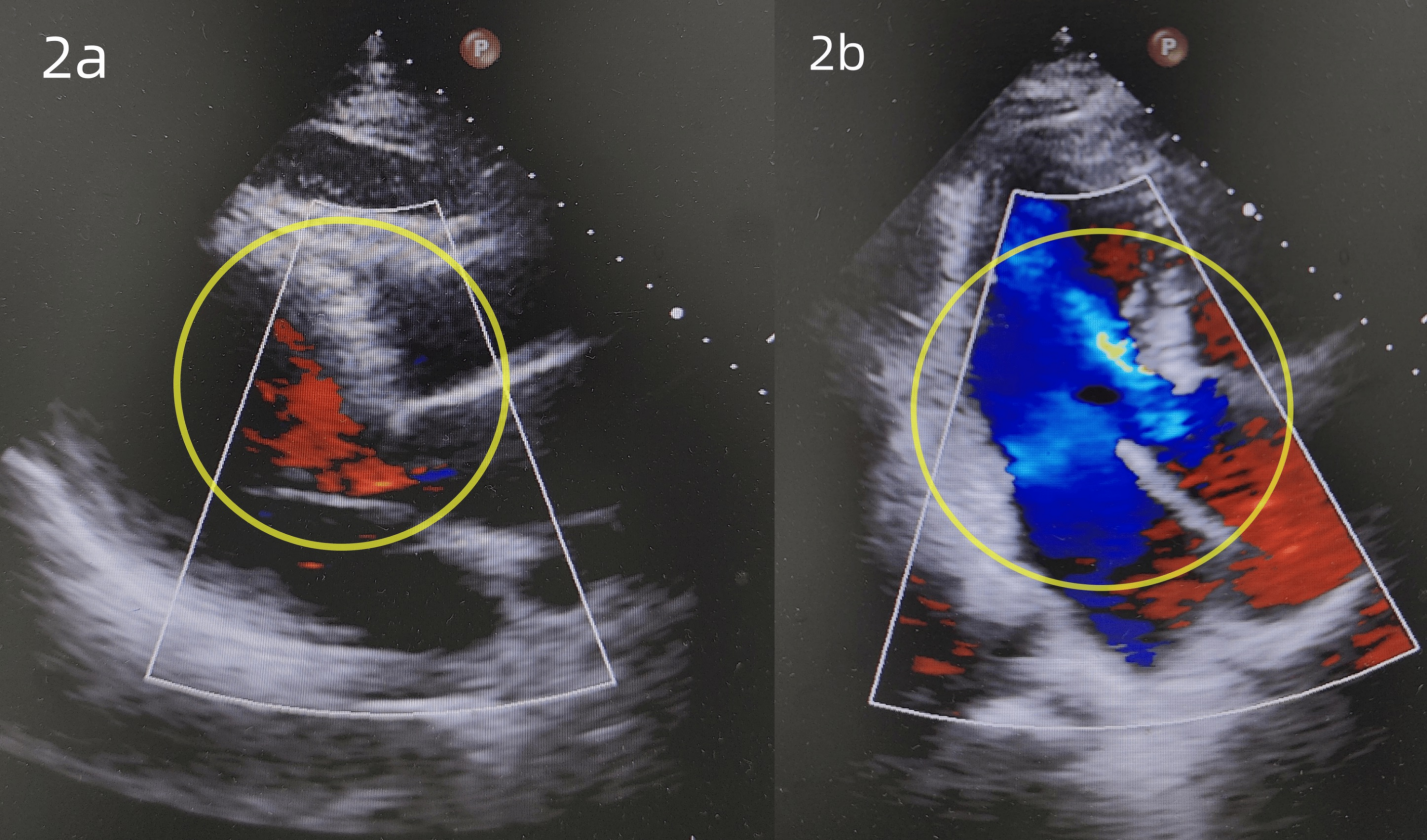
Cardiac ultrasound. (a) The yellow circle indicates left heart chamber enlargement. (b) The yellow circle indicates aortic dilation.

A 61-year-old male patient was admitted after having found a retroperitoneal mass 20 days ago. The patient came to our outpatient department with abdominal bloating and a low-grade fever, and an abdominal CT indicated a giant retroperitoneal mass, leading to hospitalization for surgical treatment. The patient had no prior medical history. Physical examination revealed abdominal distension, a soft abdomen, and a soft mass that could be felt, approximately 30*25 cm in size, in the right abdomen, with moderate mobility, no obvious tenderness or rebound tenderness, and no other significant abnormalities. Relevant auxiliary examinations were completed: enhanced CT of the entire abdomen showed a giant retroperitoneal mass with surrounding tortuous vascular shadows (Figure 1). Blood routine examination showed hemoglobin: 114 g/L (reference range: 130-175 g/L). Cardiac ultrasound indicated left heart chamber enlargement and aortic dilation (Figures 2a, 2b). Coagulation function, liver and kidney function, and the electrocardiogram showed no significant abnormalities.

**Figure 3:**
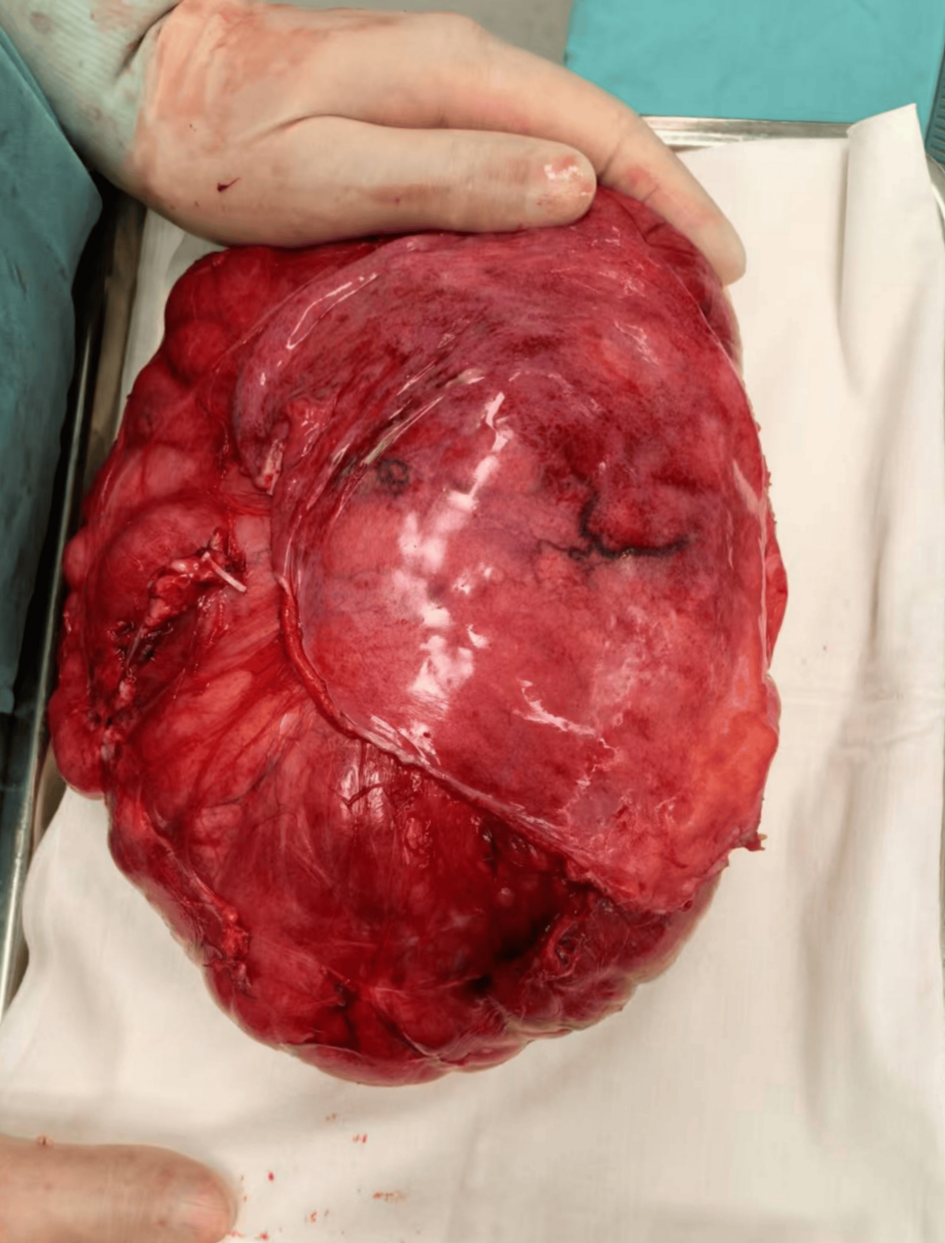
The resected retroperitoneal tumor

**Figure 4:**
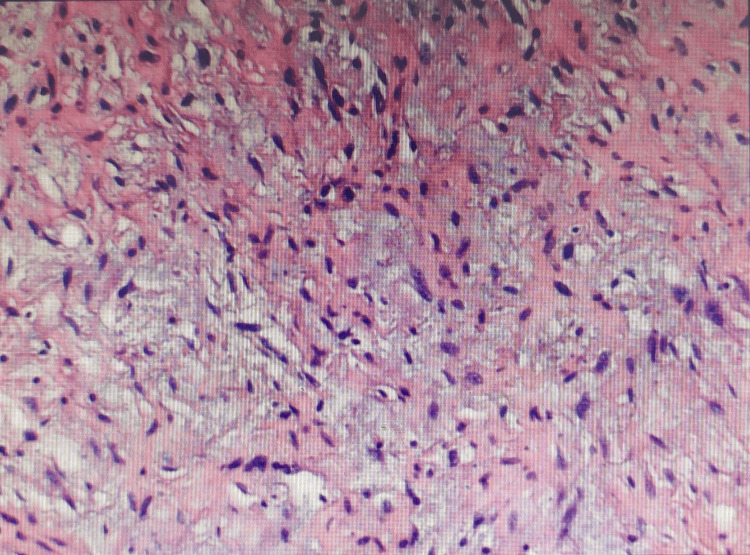
The postoperative pathology confirmed dedifferentiated liposarcoma

After a comprehensive evaluation of the patient's condition and exclusion of surgical contraindications, a retroperitoneal tumor resection was performed under general anesthesia. A midline abdominal incision of approximately 25 cm was made, revealing a small amount of clear ascites in the abdominal cavity, with the tumor located in the right lower abdomen, measuring approximately 30*25*18 cm, solid, encapsulated, with moderate mobility, and visible tortuous feeding vessels on the tumor surface. The ascending colon was pushed to the mid-abdomen by the tumor. The peritoneum was incised during the procedure to expose the tumor, and the feeding vessels were ligated to avoid damaging the tumor capsule, ensuring complete tumor resection (Figure 3). Intraoperative blood loss was 50 ml, and the surgery lasted for 2 hours. Postoperatively, the patient received fluid replacement, anti-infection treatment, gastric protection, analgesia, and dressing changes. The patient recovered well and was discharged four days after surgery. The postoperative pathological report indicated spindle cell sarcoma, which, combined with immunohistochemical staining results and molecular testing, was consistent with dedifferentiated liposarcoma (Figure 4). Immunohistochemistry results were as follows: S100 (-), SOX10 (-), H3k27me3 (+++), SMA (-), CD34 (+++), Ki67 (approximately 20%+), Desmin (scattered weak+), and ALK (-). MDM2 gene abnormality testing showed amplification of the MDM2 gene detected in the tested cells, with polyploidy of chromosome 12.

**Figure 5:**
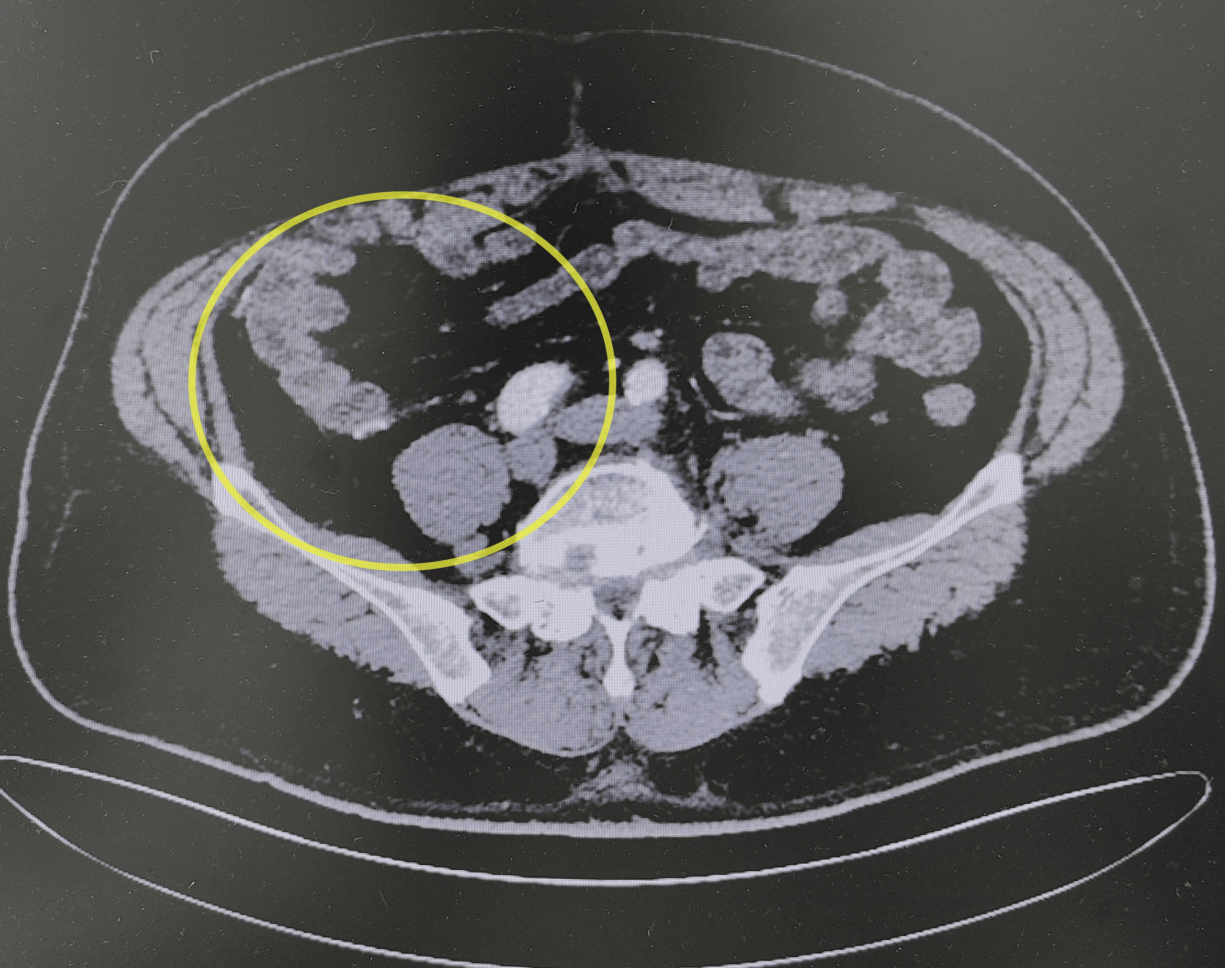
The follow-up CT of the abdomen showed postoperative changes in the right abdomen, with no recurrence or metastasis observed

Four months after surgery, a follow-up CT of the abdomen showed postoperative changes in the right abdominal cavity, with no recurrence or metastasis observed (Figure 5). The patient reported no significant discomfort and was advised to have regular follow-ups.

## Discussion

Soft tissue sarcomas are extremely rare malignant tumors, making up less than 1% of adult cancers [6]. Since these tumors originate from the mesoderm and most arise from connective tissue, muscle tissue, adipose tissue, nerve tissue, and vascular lymphatic tissue, soft tissue sarcomas exhibit considerable heterogeneity [7]. Liposarcoma is one of the most common soft tissue malignancies in adults. Retroperitoneal liposarcoma is the most common primary malignant tumor in the retroperitoneum, followed by leiomyosarcoma and malignant fibrous histiocytoma [8]. Primary retroperitoneal liposarcoma can originate from normal adipose tissue in the retroperitoneal space, accounting for 0.07% to 0.2% of all tumors, with an average age of onset of 40 to 60 years and no significant gender difference in incidence [9]. In 2013, the WHO classified liposarcoma into five types [10]: (1) well-differentiated liposarcoma; (2) dedifferentiated liposarcoma; (3) myxoid liposarcoma; (4) pleomorphic liposarcoma; (5) nonspecific liposarcoma.

Surgery is currently the main treatment for liposarcoma, but due to the presence of a fake capsule composed of cellular debris, inflammatory cells, and potential tumor tissue surrounding liposarcoma [7], most liposarcomas tend to behave aggressively, with risks of recurrence and metastasis even after surgical resection. Hematogenous metastasis to the lungs is the most common, while lymph node metastasis is relatively rare. Different subtypes of liposarcoma exhibit different oncological and histological behaviors. Research by Ghadimi et al. indicates that well-differentiated liposarcoma has a lower grade and lower metastatic potential [11], while myxoid/round cell liposarcoma has a higher grade and higher metastatic potential. Due to the difficulty in achieving negative surgical margins in retroperitoneal liposarcoma, the local recurrence rate after simple surgical resection is relatively high [12].

Surgical resection methods for retroperitoneal liposarcoma mainly include radical resection, palliative resection, debulking surgery, and tumor biopsy. Due to the high recurrence rate of liposarcoma, it is important to ensure that the resection margins are as far from the visible tumor boundaries as possible and to attempt to completely remove the pseudocapsule of the tumor. In cases where the tumor invades surrounding organs, one or more organs may need to be resected to ensure complete tumor removal, with unilateral nephrectomy being the most common. For recurrent liposarcoma, surgical resection is also recommended whenever possible. Repeated surgeries can lead to severe intra-abdominal adhesions and loss of normal anatomical structure, making it extremely challenging for the surgeon. The presence of small satellite lesions may be one reason for the high recurrence of retroperitoneal liposarcoma. For patients with multiple recurrences, organ resection may increase the thoroughness of resection, with common organs removed, including the kidneys, spleen, colon, and duodenum [13].

Recent studies have found that there is an amplification phenomenon of chromosome 12q13-15 in well-differentiated and dedifferentiated liposarcomas, with the mouse double minute 2 (MDM2) gene being considered a representative diagnostic gene [14]. Research by Bill et al. confirmed that the number of MDM2 amplifications is related to the recurrence time of dedifferentiated liposarcoma [15]. In patients receiving systemic therapy, the number of MDM2 amplifications is associated with overall survival. Furthermore, amplification of the MDM2 gene leads to the ubiquitination and degradation of the p53 gene, and MDM2 gene inhibitors are also considered potential targeted drugs. The small molecule MDM2 inhibitor SAR405838 has been shown to restore the p53 pathway and ultimately lead to apoptosis, making it a potential therapeutic agent for liposarcomas carrying MDM2 mutations [16]. This may help for future treatment if recurrence occurs.

Preoperative external beam radiotherapy can be used for liposarcoma patients, with a total dose of 50.0 to 50.6 Gy, conventionally divided into 25 to 28 sessions, each 1.8 to 2.0 Gy, completed within 5 to 6 weeks. Compared to surgery alone, surgery combined with preoperative external beam radiotherapy can improve local control rates [17]. A study in the American Cancer Database confirmed that the prolonged survival time of 1,908 patients with retroperitoneal liposarcoma who underwent surgical treatment was related to neoadjuvant radiotherapy, with a median survival time extended by 129.2 months. In patients with tumor invasion of adjacent organs, those receiving neoadjuvant radiotherapy had significantly longer median survival times compared to those who did not receive it [13]. In terms of targeted drug therapy, recent years have seen the emergence of a number of targeted drugs acting on liposarcoma, such as multi-target tyrosine kinase inhibitors and vascular endothelial growth factor receptor (VEGFR) inhibitors, as basic research advances and understanding of tumor biological behavior deepens.

The recurrence rate after surgical resection of retroperitoneal liposarcoma is high, mainly due to local recurrence, with distant metastasis occurring rarely. Moreover, as the number of recurrences increases, the interval between recurrences tends to shorten. Factors contributing to postoperative recurrence may include: (1) the tumor's large size and close relationship with surrounding organs and tissues, making complete resection challenging; (2) the tumor's capsule being a pseudocapsule formed by continuous pressure from the tumor on its outer tissue, often resulting in residual tumor tissue after surgical resection; (3) multifocal growth of the tumor, making it easy to miss small tumor foci during surgery. The overall 5-year survival rate for patients with retroperitoneal tumors ranges from 40% to 60%, with 3-year survival rates of 73% and 43% for patients with complete and incomplete resection, respectively [18]. Active postoperative follow-up is key to early detection of tumor recurrence and timely treatment. Patients should undergo CT or ultrasound re-examinations every six months within three years after surgery, especially for poorly differentiated tumor types, with particular attention to follow-ups in the first year to detect tumor recurrence as early as possible.

The limitation of this case lies in the lack of further molecular biological analysis of the tumor tissue and the relatively short follow-up time, with long-term survival data yet to be completed. Second, the patient's preoperative MRI evaluation is missing, and the MDM2 amplification findings lack detailed quantitative analysis. Without these essential diagnostic elements, it becomes difficult to determine whether this represents pure dedifferentiated liposarcoma, well-differentiated liposarcoma, or malignant transformation from well-differentiated to dedifferentiated components, limiting the case's educational value for identifying similar patients. Nevertheless, this case provides a detailed description of the successful surgical resection of a giant retroperitoneal dedifferentiated liposarcoma and the patient's smooth recovery, vividly illustrating the surgical skills and perioperative management strategies required to handle such challenging cases.

## Conclusions

This case report provides a detailed description of the treatment process for a patient with a giant retroperitoneal dedifferentiated liposarcoma who underwent radical surgical resection and ultimately recovered and was discharged. Even with the tumor's large size and complicated anatomy, safe and effective tumor resection was achieved through careful preoperative assessment and consistent perioperative care, with no serious complications occurring. Future research should focus on further exploring the role of neoadjuvant therapy (especially targeted therapy for DDLPS, such as MDM2 inhibitors) in reducing tumor size and promoting resectability. Additionally, long-term survival follow-up and quality of life assessments should be conducted, along with molecular typing studies to guide individualized precision treatment. We believe radical resection remains the mainstay of treatment for giant DDLPS. This case provides valuable surgical experience for managing giant retroperitoneal liposarcoma and shows how important a surgery-focused comprehensive treatment is in improving the quality of life for patients.
